# National Burden and Trend of Cancer in Ethiopia, 2010–2019: a systemic analysis for Global burden of disease study

**DOI:** 10.1038/s41598-022-17128-9

**Published:** 2022-07-26

**Authors:** Atalel Fentahun Awedew, Zelalem Asefa, Woldemariam Beka Belay

**Affiliations:** 1 Department of Surgery, SoM, Addis Ababa University, Addis Ababa, Ethiopia; 2 Department of Clinical Oncology, SoM, Addis Ababa University, Addis Ababa, Ethiopia

**Keywords:** Cancer, Oncology

## Abstract

Over the last two decades, we have tracked the national burden of cancer and its trends in Ethiopia, providing estimates of incidence, death, and disability adjusted life years. In Ethiopia, there were an estimated 53,560 (95% UI 52,480–55,540) new incident cases, 39,480 deaths (95% UI 32,640–46,440), and 1.42 million (95% UI 1.16–1.68) DALYs of cancer 2019. Cancer incidence, death, and DALYs counts increased by 32% (95% UI 11–55%), 29% (95% UI 12–44%), and 19% (95% UI − 2 to 44%) between 2010 to 2019, respectively, while age-standardised incidence, death, and DALYs rates increased by 5% (95% UI − 7 to 18%), 2% (95% UI − 9 to 14%), and − 2% (95% UI − 15 to 12%) respectively. In 2019, the leading incidence cases were leukemia, cervical cancer, breast cancer, colon and rectum cancer, and stomach cancer, while leukemia, breast cancer, cervical cancer, and stomach cancer were the most common killer cancers in Ethiopia. According to the findings of this study, tobacco-related cancers such as pancreatic, kidney, and lung cancer have increased in Ethiopian females over the last decade, while genitourinary cancer has increased in Ethiopian males. Another significant finding was that infection-related cancers, such as stomach cancer and Hodgkin lymphoma, have been rapidly declining over the last decade.

## Introduction

Cancer is a newly evolved noncommunicable global disease burden that accounts for a significant portion of global morbidity, mortality, and economic loss. Cancer is the first or second leading cause of premature death in 134 of the world's 183 countries, and it ranks third or fourth in 45 more for people under the age of 70^1–3^. According to WHO, cancer caused 4.5 million (29.8 percent) of the 15.2 million premature deaths from noncommunicable diseases worldwide in 2016, while cardiovascular diseases caused 6.2 million (40.8 percent)^1^. Cancer caused approximately 24 million new cases, 10.0 million deaths, and 250 million DALYs globally in 2019^4^. Because of demographics, epidemiological transitions, advanced diagnostic tools, and screening programs, these global records revealed that the burden of cancer is increasing while the burden of infectious diseases is decreasing. Because of the rapid rise in cancer cases, the United Nations (UN) Sustainable Development Goals (SDGs) include cancer burden reduction as a goal. According to 3.4, noncommunicable disease premature mortality should have been reduced by one-third through prevention and treatment, as well as promotion of mental health and well-being^5^. Global integrated and cooperative efforts on prevention and control of cancer lead by the WHO initiative focused breast Cancer^6^, Cervical cancer^7^, childhood cancer^8^, and Third United Nations high Level meeting on NCDs including cancer^9^. Cancer incidence is higher in developing countries, which may be due to epidemiological transitions as well as ineffective preventive and control health policies. Ethiopia has created a National Cancer Control Plan for 2016–2020, with the goal of promoting cancer prevention, early detection, improved diagnosis and treatment, palliative care, cancer surveillance, registration, and research^10^. The Global Burden of Diseases, Injuries, and Risk Factors Study 2019 (GBD 2019) provides the most recent and concise evidence of cancer burden and trends in terms of incidence, mortality, and DALYs^11^.

## Result

In 2019, there were an estimated 53,560 new incident cases of cancer in both sexes (95% UI 52,480–55,540), with an age-standardised incidence rate of 104.3 (95% UI 98.1–113.3) per 100,000 in Ethiopia. In 2019, cancer accounted for 39,480 deaths (95% UI 32,640–46,440) in both sexes, with an age-standardised death rate of 87.5 (71.6–105) per 100,000. In 2019, there were 1.42 million (95% UI 1.16–1.68) DALYs in both sexes in Ethiopia, with age-standardised rates of 2300 (95% UI 1900–2700) per 100,000. Year life loss (YLL) accounted for more than 98 percent of DALYs, with YLD accounting for the remainder (Table 1). Between 2010 and 2019, cancer incident cases, death counts, and DALYs counts increased by 32% (95% UI 11–55%), 29% (95% UI 12–44%), and 19% (95% UI − 2 to 44%), respectively, while incident, death, and DALY rates changed by 5% (95% UI − 7 to 18%), 2% (95% UI − 9 to 14%), and − 2% (95% UI 15–12%), respectively (Table 2). Between 2010 and 2019, the absolute number of years lived with disability (YLD) and years of life lost (YLL) increased by 36% (95% UI 12–64%) and 19% (95% UI − 24.4%), respectively. However, the age standardized rate of YLD increased by 9% (95% UI − 4 to 24%), while the age standardized rate of YLL decreased by − 2% (95% UI 15–12%). We made comparisons with neighboring countries as well as at the global level (Table 3).

**Table 1:** National incidence, deaths and DALYs of cancer in Ethiopia, 2019.

Cancer type	Incidence case			Age-standardised incidence rate			Death counts			Age standardised death rate			DALYs counts			Age-standardised DALYs rate		
	2019	95% UI		2019	95% UI		2019	95% UI		2019	95% UI		2019	95% UI		2019	95% UI	
Bladder cancer	1060	740	1380	2.9	2	3.7	850	570	1110	2.5	1.7	3.3	18,470	12,520	24,100	47	31.7	61.5
Brain and central nervous system cancer	1380	1070	2040	1.9	1.5	2.5	1130	890	1590	1.7	1.3	2.2	59,910	44,210	91,080	65	51.5	90.3
Breast cancer	5900	4640	7420	12.5	10.1	15.3	4110	3300	4960	9.7	8	11.6	129,580	101,090	161,690	251.9	201.6	306.7
Cervical cancer	6570	4470	10,640	12.1	8.4	19.3	3870	2680	6290	8.2	5.7	13.3	133,580	90,860	219,610	244.9	169	398.1
Colon and rectum cancer	3200	2400	4460	7.7	5.8	10.7	2850	2130	4000	7.3	5.5	10.4	79,050	58,530	109,670	168.6	124.8	236
Esophageal cancer	1080	850	1500	2.7	2.1	3.7	1120	870	1570	2.9	2.2	4	29,770	22,790	41,210	67.1	51.8	93.4
Gallbladder and biliary tract cancer	520	390	670	1.4	1	1.8	520	390	670	1.4	1.1	1.8	12,740	9480	16,360	30.1	22.5	38.2
Hodgkin lymphoma	650	490	880	0.8	0.6	1.1	470	370	640	0.7	0.5	0.9	24,450	18,500	34,140	26.9	21	36.3
Kidney cancer	740	430	1010	1.5	0.9	2.2	520	290	740	1.3	0.7	1.8	15,940	9490	22,070	30.1	16.9	43.5
Larynx cancer	410	330	550	1	0.8	1.3	380	300	510	0.9	0.7	1.3	10,870	8590	14,420	23.6	18.8	31.5
Leukemia	8310	4270	12,440	9.5	5.3	13.4	5270	2910	7250	7.7	4.5	10.9	305,610	159,470	444,030	290.1	163.5	397.2
Lip and oral cavity cancer	1170	830	1530	2.5	1.8	3.3	780	530	1010	1.8	1.3	2.4	24,130	16,440	31,410	48.1	32.6	62.6
Liver cancer	1160	930	1480	2.7	2.2	3.5	1230	970	1550	3	2.4	3.8	36,980	29,050	46,870	69.8	55.1	88.6
Malignant skin melanoma	290	220	400	0.5	0.4	0.7	230	170	310	0.5	0.4	0.6	8260	6390	11,770	13.8	10.5	19
Mesothelioma	110	40	350	0.3	0.1	0.8	90	30	290	0.2	0.1	0.7	2640	990	8100	5.3	2	17.2
Multiple myeloma	350	220	470	0.9	0.6	1.2	330	210	420	0.9	0.5	1.1	8380	5300	10,930	19.3	12.2	25
Nasopharynx cancer	530	340	700	1	0.6	1.4	510	320	670	1	0.6	1.4	18,450	11,650	24,530	31.8	19.9	42.1
Non-Hodgkin lymphoma	480	350	690	0.9	0.7	1.4	490	350	690	1	0.7	1.5	17,280	12,670	23,620	28.3	20.4	40
Non-melanoma skin cancer	2150	1730	2690	4.8	3.9	5.8	210	100	290	0.7	0.3	0.9	4620	2250	6120	11.4	5.3	15.1
Other malignant neoplasms	4790	3990	5770	7.4	6.1	8.6	4330	3660	5130	7.2	6	8.2	214,780	171,450	268,160	245.3	206.3	289.5
Other pharynx cancer	160	100	230	0.3	0.2	0.5	150	100	220	0.3	0.2	0.5	4620	3120	6780	9.5	6.3	13.9
Ovarian cancer	1300	720	2120	2.6	1.4	4.1	910	490	1500	2	1.1	3.3	29,770	16,110	49,910	57.5	30.8	94.5
Pancreatic cancer	570	380	820	1.5	1	2.1	600	410	870	1.6	1.1	2.3	14,860	9860	21,500	34.6	23.1	50.2
Prostate cancer	2570	1350	4300	7.5	4	12.3	2290	1210	3740	7.1	3.8	11.3	43,410	22,750	72,730	121.3	64.2	200.3
Stomach cancer	2580	2100	3230	6.2	5.1	7.7	2640	2160	3370	6.6	5.4	8.3	73,970	59,220	95,580	155.4	126.2	196.8
Testicular cancer	270	180	440	0.2	0.2	0.4	90	50	110	0.1	0.1	0.1	5110	3250	6670	5	3	6.5
Thyroid cancer	2500	1790	3370	4	3	5.3	850	630	1090	1.9	1.4	2.5	28,680	21,690	37,280	50.7	37.6	65.3
Tracheal, bronchus, and lung cancer	2170	1510	2920	5.6	3.9	7.5	2310	1600	3130	6.1	4.2	8.3	55,500	38,360	75,110	131.8	91	178.9
Uterine cancer	590	420	870	1.4	1	2.1	350	250	520	0.9	0.6	1.4	9060	6460	13,700	20.8	14.8	31.5
Total	53,560	52,480	55,540	104.3	98.1	113.3	39,480	32,640	46,440	87.5	71.6	106	1,420,000	1,160,000	1,680,000	2300	1900	2700

**Table 2:** Percentage changes of national incidence cases, deaths and DALYs in Ethiopia from 2010 to 2019.

Cancer type	Incidence case change (%)			ASIR change (%)			Death counts change (%)			ASDR change (%)			DALYs counts change (%)			Age standardised DALYs rate change (%)		
	Value	95% UI		Value	95% UI		Value	95% UI		Value	95% UI		Value	95% UI		Value	95% UI	
Bladder cancer	46	24	75	9	− 7	30	38	16	63	3	− 12	21	33	12	58	1	− 14	19
Brain and central nervous system cancer	23	− 7	65	1	− 18	25	24	− 6	63	1	− 17	23	17	− 15	64	− 2	− 24	27
Breast cancer	60	28	99	19	− 3	42	47	21	74	10	− 7	27	43	14	77	7	− 12	28
Cervical cancer	33	1	73	1	− 22	27	28	− 2	63	− 2	− 24	22	23	− 7	60	− 6	− 28	20
Colon and rectum cancer	62	33	92	21	− 1	42	56	30	83	16	− 4	36	54	27	81	15	− 5	35
Esophageal cancer	24	4	46	− 6	− 20	11	25	4	48	− 5	− 20	12	21	− 1	45	− 8	− 24	10
Gallbladder and biliary tract cancer	30	9	57	− 1	− 16	18	30	10	56	− 2	− 16	18	25	4	52	− 5	− 20	15
Hodgkin lymphoma	13	− 8	43	− 14	− 28	5	6	− 12	31	− 18	− 31	− 3	4	− 16	32	− 19	− 33	− 1
Kidney cancer	60	33	99	26	6	56	55	29	91	21	1	50	43	15	79	17	− 4	45
Larynx cancer	20	− 2	49	− 9	− 25	12	17	− 5	44	− 11	− 28	9	15	− 8	43	− 13	− 30	7
Leukemia	− 3	− 32	38	− 11	− 31	11	3	− 23	38	− 8	− 26	12	− 6	− 33	37	− 15	− 36	10
Lip and oral cavity cancer	47	23	76	10	− 6	31	39	18	66	5	− 11	25	37	13	66	3	− 14	23
Liver cancer	30	8	57	1	− 16	19	31	9	57	1	− 15	21	21	− 2	48	− 2	− 19	19
Malignant skin melanoma	44	11	83	8	− 14	32	34	5	67	2	− 18	23	31	− 2	69	− 1	− 22	24
Mesothelioma	36	5	72	4	− 18	28	38	9	74	5	− 15	30	33	2	71	1	− 20	28
Multiple myeloma	42	16	70	7	− 12	28	40	14	66	6	− 13	24	36	10	65	3	− 16	23
Nasopharynx cancer	27	6	51	− 4	− 19	13	27	6	51	− 4	− 19	13	24	3	52	− 6	− 21	13
Non-Hodgkin lymphoma	46	17	77	10	− 10	32	41	12	70	9	− 12	33	33	2	64	6	− 16	28
Non-melanoma skin cancer	35	32	39	0	− 3	3	55	32	83	14	− 3	33	47	23	76	10	− 6	31
Other malignant neoplasms	39	19	70	5	− 9	21	29	8	54	1	− 12	14	24	− 1	56	0	− 16	19
Other pharynx cancer	47	19	78	11	− 9	33	46	20	74	10	− 9	31	44	19	74	8	− 11	30
Ovarian cancer	67	16	134	24	− 10	68	61	20	117	21	− 8	59	60	16	123	20	− 11	63
Pancreatic cancer	59	32	99	20	0	51	59	32	102	20	− 1	52	56	29	97	18	− 3	49
Prostate cancer	57	28	90	16	− 4	40	47	20	77	9	− 10	31	43	15	74	8	− 12	31
Stomach cancer	11	− 4	28	− 16	− 26	− 4	12	− 3	27	− 15	− 26	− 4	8	− 8	26	− 18	− 30	− 6
Testicular cancer	67	− 2	180	33	− 12	104	47	13	92	11	− 12	40	45	10	91	11	− 14	45
Thyroid cancer	54	17	107	14	− 8	44	29	8	53	− 1	− 15	14	24	− 1	54	− 5	− 20	14
Tracheal, bronchus, and lung cancer	38	13	70	4	− 16	29	38	11	72	4	− 17	30	37	10	71	3	− 17	27
Uterine cancer	53	22	91	16	− 7	43	37	10	68	5	− 15	27	32	4	65	1	− 19	25
Total	32	11	55	5	− 7	18	29	12	44	2	− 9	14	19	− 2	44	− 2	− 15	12

**Table 3:** Percentage changes of incidence cases, deaths and DALYs in global and neighbor countries from 2010 to 2019.

	Change of incidence cases (%)			Change of ASIR (%)			Change of deaths counts (%)			Change of ASDR (%)			Change of DALYs (%)			Change of ASDALYsR (%)		
	Value	95% UI		Value	95% UI		Value	95% UI		Value	95% UI		Value	95% UI		value	95% UI	
Global	26	20	32	− 1	− 6	3	21	14	28	− 6	− 11	− 1	16	9	23	− 7	− 12	− 1
Djibouti	58	26	99	6	− 12	28	54	23	92	2	− 14	23	43	10	82	0	− 19	24
Eritrea	35	14	62	3	− 12	20	32	11	56	0	− 13	17	27	5	53	− 1	− 16	17
Ethiopia	32	11	55	5	− 7	18	29	12	48	2	− 9	14	19	− 2	44	− 2	− 15	12
Kenya	40	21	64	2	− 11	17	36	18	56	− 1	− 12	12	31	12	53	− 4	− 16	11
Somalia	32	10	58	− 3	− 18	15	30	9	55	− 4	− 17	13	28	6	54	− 5	− 20	13
South Sudan	16	− 2	38	0	− 14	17	16	− 3	40	− 2	− 17	17	10	− 9	33	− 2	− 18	19
Sudan	38	17	66	11	− 5	30	30	11	55	5	− 9	23	26	3	54	2	− 14	23

*ASIR* age-standardised incidence rate, *ASDR* age-standardised death rate, *ASDALYsR* age-standardised DALYs rate.

### Burden of cancer type

In Ethiopia, the five most common cancer incidence cases were leukemia (8310 [95% UI 4270–12440]), cervical cancer (6570 [95% UI 4470–10640]), breast cancer (5900 [95% UI 4640–7420]), colon and rectum cancer (3200 [95% UI 2400–4460]), and stomach cancer (2580 [95% UI 2100–3230]). In 2019, the highest age-standardised incidence rate was observed in breast (12.5 [95% UI 10.1–15.3]), cervical cancer (12.1 [95% UI 8.4–19.3]), leukemia (9.5 [95% UI 5.3–13.4]), CRC (7.7 [95% UI 5.8–10.7]), and prostate cancer 7.5 (95% UI 4–12.3) per 100,000, while the lowest age-standardised incidence rate was noted in malignant skin melanoma 0.5 (95% UI 0.4–0.7), mesothelioma 0.3 (95% UI 0.1–0.8), other pharynx cancer 0.3 (95% UI 0.2–0.5), and testicular cancer 0.2 (95% UI 0.19–0.4) per 100,000 (Table 1). From 2010 to 2019, the highest change of age-standardised incidence rates was recorded in testicular cancer 33% (95% UI − 12 to 104%), kidney 26% (95% UI 6–56%), ovarian 24% (95% UI − 10 to 68%), and CRC 21% (95% UI − 1 to 42%), while the lowest changes of age- standardised incident rates were observed in leukemia-11% (95% UI − 31 to 11%), Hodgkin lymphoma-14% (95% UI − 28 to 5%), and stomach cancer-16% (95% UI − 26 to − 4%) (Table 2).

Leukemia (5270 [95% UI 2910–7250]), breast cancer (4110 [95% UI 3300–4960]), cervical cancer (3870 [95% UI 2680–6290]), CRC (2850 [95% UI 2130–4000]), and stomach cancer (2640 [95% UI 2160–3370] were the four most lethal cancers in Ethiopia in 2019. In 2019, breast cancer 9.7 (95% UI 8–11.6), cervical cancer 8.2 (95% UI 5.7–13.3), leukemia 7.7 (95% UI 4.5–10.9), and CRC 7.7 (95% UI 5.5–10.4) per 100,000 had the highest age-standardised death rates in Ethiopia (Table 1). From 2010 to 2019, the highest percentage change of death counts was observed in ovarian cancer 61% (95% UI 20–117%), pancreatic cancer 59% (95% UI 32–102%), CRC 56% (95% UI 30–83%), and non-melanoma skin cancer 55% (95% UI 32–83%), while the lowest death count changes were seen in stomach cancer 12% (95% UI − 3 to 27%), Hodgkin's lymphoma 6% (95% UI − 12 to 31%), and leukemia 3% (95% UI − 23 to 38%) (Table 2).

In 2019, leukemia, breast cancer, cervical cancer, colorectal cancer, and stomach cancer had the highest age-standardised DALYs rate (Table 1). From 2010 to 2019, the highest DALYs count changes were observed in ovarian cancer 60% (95% UI 16–123%), pancreatic cancer 56% (95% UI 27–81%), colon and rectum cancer 54% (95% UI 27–81%), non-melanoma skin cancer 47% (95% UI 23–76%), while lowest change of DALYs counts documented in stomach cancer 8% (95% UI − 8 to 26%), Hodgkin's lymphoma 4% (95% UI − 16 to 32%), and leukemia-6% (95% UI − 33 to 37%). The highest age-standardised rate of DALYs change from 2010 to 2019 has been seen in pancreatic cancer 18% (95% UI − 5 to 35%), and colon and rectum cancer 15% (95% UI − 5 to 35%), while the lowest changes have been seen in Hodgkin's lymphoma-2% (95% UI − 24 to 27%), and leukemia-19% (95% UI − 33 to − 1%) (Table 2).

### Burden of cancer in female

**Figure 1:**
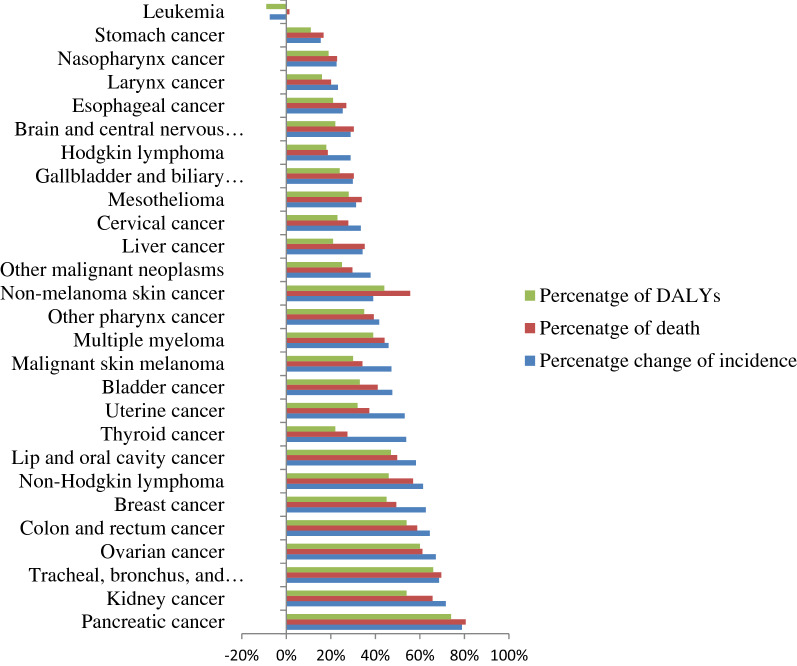
Percentage change of incidence, death, and DALYs in each cancer type among females in Ethiopia from 2010 to 2019.

In 2019, the leading incident cases of cancer in females were cervical cancer (6570 [95% UI 4470–106400], breast cancer (5450 [95% UI 4210–6860]), leukemia (3980 [95% UI 1890–6390]), thyroid (1990 [95% UI 1360–2790]), and CRC (1440 [95% UI 1020–2120]), while the lowest incident cases were larynx cancer (60 [95% UI 50–80]), mesothelioma (60 [95% UI 20–90]) and other pharynx cancer (50 [95% UI 40–80]). Cervical cancer 24.6 (95% UI 17.1–39.2), breast cancer 23 (95% UI 18.4–28.1), leukemia 8.7 (95% UI 4.9–12.4), and CRC 7 (95% UI 5–10.2) per 100,000 Ethiopian females had the highest age-standardised incidence rate in 2019. From 2010 to 2019, the highest percentage of change of incidence cases in females was observed in pancreatic 79% (95% UI 42–128%), kidney cancer 72% (95% UI 33–115%), tracheal, bronchus, and lung cancer 69% (95% UI 36–114), ovarian cancer 67% (95% UI 16–134%), and CRC 64% (95% UI 32–104), while the lowest change was observed in stomach 15% (95% UI − 7 to 44%) and leukemia-7% (95% UI − 39 to 43%) (Fig. 1).

Stomach cancer, leukemia, larynx cancer, nasopharynx cancer, esophageal cancer, Hodgkin's lymphoma, gallbladder and biliary tract cancer, and mesothelioma have decreased age-standardised incidence rate from 2010 to 2019, ranging from − 1 to − 14%, and other malignant cancers have seen an increment in age-standardised incidence rate, ranging from 1 to 30%.

**Figure 2:**
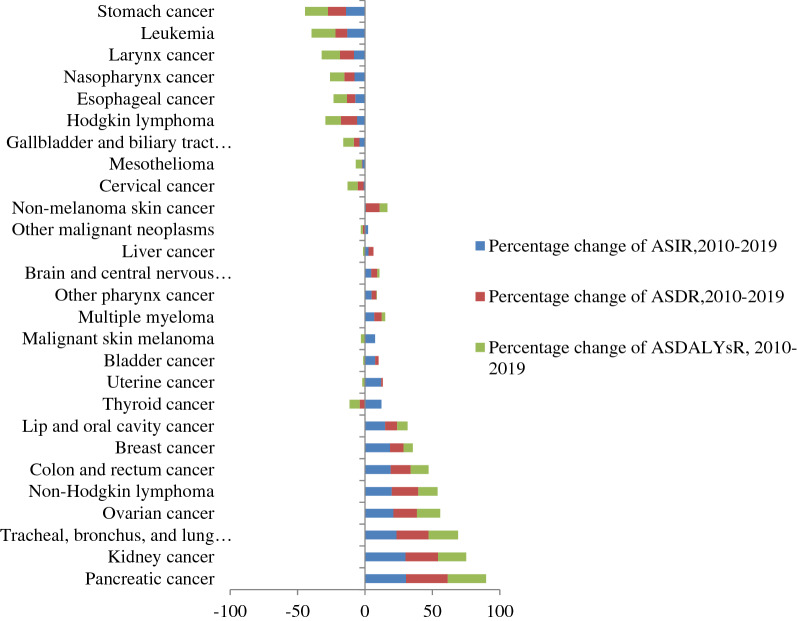
Percentage change of age-standardised rate of incidence, death, and DALYs in each cancer type among females in Ethiopia from 2010 to 2019.

In 2019, the leading causes of cancer related mortality were cervical cancer 3870 (95% UI 2680–6290), breast cancer 3700 (95% UI 2970–4510), leukemia 2150 (95% UI 1260–3060), CRC 1270 (95% UI 900–1870), and stomach cancer 1230 (95% UI 950–2030), while the lowest causes of death were non-melanoma skin cancer 60 (95% UI 30–90), mesothelioma 50 (95% UI 20–70), and other pharynx cancer 50 (95% UI 30–70). From 2010 to 2019, the highest percentage change of death was observed in pancreatic cancer at 81% (95% UI 43–128), tracheal, bronchus, and lung cancer 70% (95% UI 39–112), kidney cancer 66% (95% UI 33–100), ovarian cancer 61% (95%), and CRC 59% (95% UI 30–93%), while the lowest change of death was seen in Hodgkin's lymphoma 19% ( 95% UI − 10 to 58%), stomach cancer 17% (95% UI − 4 to 43%), and leukemia 1% (95% UI − 28 to 46%) (Fig. 2).

### Burden of cancer in males

**Figure 3:**
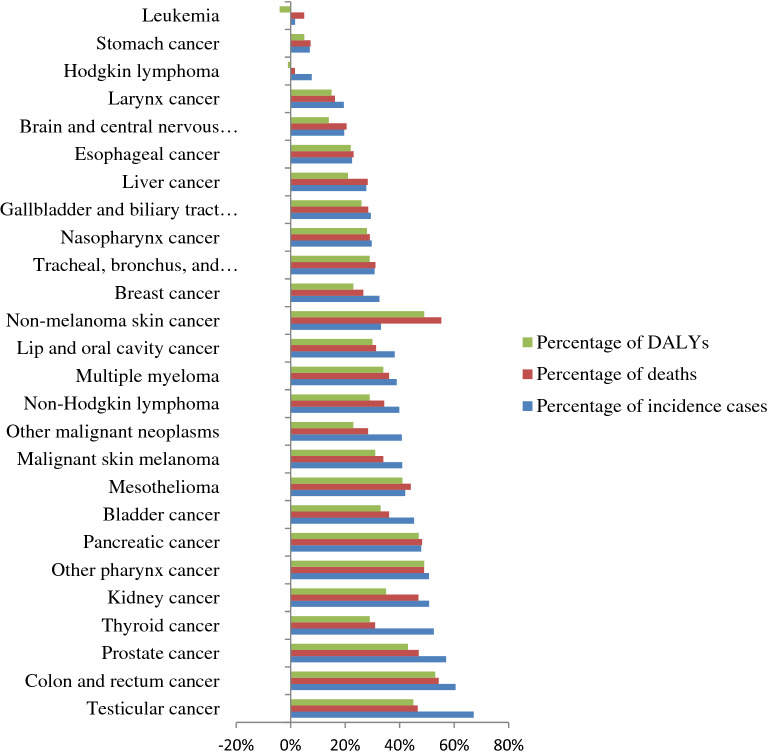
Percentage change of incidence, death, and DALYs in each cancer type among males in Ethiopia from 2010 to 2020.

In 2019, the leading causes of incident cases of cancer among males were leukemia (4330 [95% UI 2110–6470]), prostate cancer (2570 [95% UI 1350–4300]), CRC (1760 [95% UI 1230–2690]), tracheal, bronchial, and lung cancer (1690 [95% UI 1160–2280]). In 2019, the highest age-standardised incidence rate was observed in prostate cancer (14.5 [95% UI 7.7–23.9]), leukemia (10.3 [95% UI 5.3–15.6]), tracheal, bronchus, and lung cancer (8.6 [95% UI 5.9–11.6]), and CRC (8.4 [95% UI 5.9–12.7]) per 100,000, while the lowest age-standardised incidence rate was observed in testicular cancer 0.5 (95% UI 0.3–0.7), other pharynx cancer (0.4 [95% UI 0.3–0.7]), and mesothelioma (0.2 [95% UI 0.1–01.3]) per 100,000. Testicular cancer (67% [95% UI 2–180]), colon and rectum cancer (61% [95% UI 19–105]), prostate cancer (57% [95% UI 28–90]), and thyroid cancer (53% [95% UI 19–103]) had the highest percentage change in incident cases from 2010 to 2019, while Hodgkins lymphoma (8% [95% UI 14–36%]), stomach cancer (7% [95% UI − 14 to 32%]), and leukemia (2% [95% UI − 29 to 51%]) had the lowest change of incident cases (Fig. 3).

**Figure 4:**
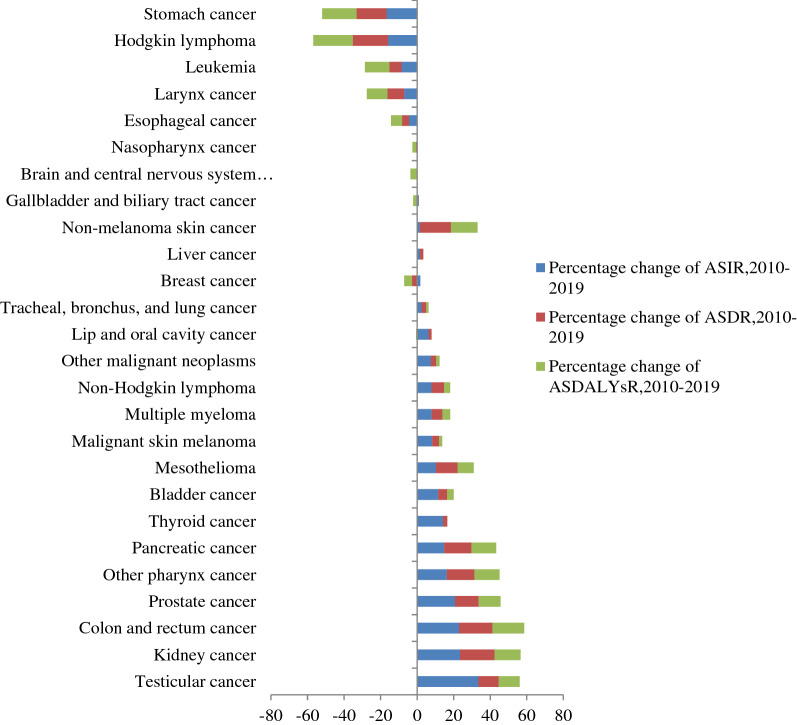
Percentage change of age-standardised rate of incidence, death, and DALYs in each cancer type among males in Ethiopia from 2010 to 2019.

In 2019, the five most lethal cancers in Ethiopian males were leukemia (3120 [95% UI 1540–4540]), prostate cancer (2290 [95% UI 1210–3740]), tracheal, bronchus, and lung cancer (1820 (95% UI 1240–2480]), colon and rectum cancer (1580 [95% UI 1090–2450]), and stomach cancer (1410 [95% UI 1050–1840]). Prostate cancer had the highest age-standardised death rate in Ethiopia in 2019, with 13.8 (95% UI 7.5–22), tracheal, bronchus, and lung cancer (9.5 [95% UI 6.5–12.90]), leukemia (9 [95% UI 4.7–14]), and colon and rectum cancer (8 [95% UI 5.5–12.3]). Kidney cancer (19% [95% UI − 14 to 67%]), colon and rectum cancer (18% [95% UI − 11 to 50%]), non-melanoma skin cancer (17% [95% UI − 4 to 41%]), other pharynx cancer (15% [95% UI − 13 to 50%]), and pancreatic cancer (15% [95% UI − 14 to 59%]) had the greatest percentage change in age-standardised death rate in males between 2010 and 2019, while decreased age-standardised death rate in males was observed in breast cancer (− 3% [95% UI − 27 to 27%]), esophageal cancer (− 4% [95% UI − 26 to 25%]), leukemia (− 7% [95% UI − 28 to 23%]), larynx cancer (− 9% [95% UI − 29 to 14%)), stomach cancer (− 16% [95% UI − 33 to 2%), and Hodgkin's lymphoma (− 19% [95% UI − 35 to 0%]) (Fig. 4).

In 2019, the leading causes of highest DALYs counts and age-standardised DALYs rates in males were leukemia, prostatic cancer, CRC, and tracheal, bronchus, and lung cancer, while the lowest DALYs counts were non-melanoma skin cancer, other pharynx cancer, and mesothelioma. From 2010 to 2019, the most significant percentage change in DALYs counts was observed in colon and rectum cancer (53% [95% UI 13–97%]), non-melanoma skin cancer (49% [95% UI 19–85%], other pharynx cancer (49% [95% UI 12–97%]), pancreatic cancer (47% [95% UI 10–103%]), testicular cancer (45% [95% UI 10–91%]), and prostate cancer (15% [95% UI:15–74%]) (Fig. 3). From 2010 to 2019, a decreased age-standardised DALYs rate was identified in lip and oral cavity cancer, nasopharynx cancer, gallbladder and biliary tract cancer, brain and central nervous system cancer, breast cancer, esophageal cancer, larynx cancer, leukemia, stomach cancer, and Hodgkin lymphoma, ranging from − 1 to − 22% (Fig. 4).

## Discussion

Between 2010 and 2019, the absolute number of cancer incidence cases, mortality, and DALYs increased significantly in Ethiopia. However, the age-standardised rate of cancer incidence, death, and DALYs shows erratic trends. From 2010 to 2019, we found that the trends in the age-standardised cancer incidence rate were fairly stable. Similarly, neighboring countries such as Djibouti, Eritrea, Kenya, Somalia, South Sudan, and Sudan have seen similar trends. However, there were contrast trend in high income countries and global^4^. Cancer is becoming more prevalent around the world, particularly in low and middle-income countries. According to WHO projections, low and middle income (LMIC) countries will bear two-thirds of the cancer burden in 2040^3^.The main reasons for the rapid rise in cancer in low and middle income countries are population growth, aging, sociodemographic, and epidemiological transitions (LMICs)^1^.

The change in the incidence of cancer cases in Ethiopia, on the other hand, was primarily driven by population growth and aging. The stable age-standardised cancer incidence rate suggests that epidemiological and sociodemographic transitions play a minor role in cancer pathogenesis in Ethiopia. In 2019, behavioral risks, metabolic, occupational exposure, and air pollutions were attributed to approximately 20% (17–26%) of cancer in Ethiopia; however, changes in overall risk factors were less than 10% between 2010 and 2019.From 2010 to 2019, the age-standardised rate of cancer death in Ethiopia increased. The findings of this study stand in stark contrast to the age-standardised cancer rates in high-income countries^12^ and global trends^4^.

The increased age-standardised cancer death rate calls into question national policy in terms of progress in treatment and management, primary prevention, and secondary prevention modality implementation. Cancer is responsible for one out of every six deaths worldwide, according to a WHO report^3^. Many global initiatives have been launched to address the cancer burden. However, global, regional, and national efforts for cancer prevention in low and middle-income countries remain insufficient and inequitable^4^. In high-income countries, a strong health-care system, a large human resource capacity, and effective primary and secondary prevention methods are responsible for lower mortality^12^ whereas the main causes of increased cancer related mortality are a lack of workforce capacity, poor cancer care infrastructure, a lack of cancer centers for diagnosis and treatment, a lack of financial security, and a lack of universal health coverage^3,13^. Despite an increase in the age-standardised death rate of overall cancer, some cancer types had decreased age-standardised death rates, such as thyroid cancer, gall bladder and biliary tract cancer, cervical cancer, Nasopharynx cancer, esophageal cancer, leukemia, larynx cancer, stomach cancer, and Hodgkin lymphoma, which ranged from − 1 to − 18% over the last one decades. The main agent for lowering mortality rates will be progress in human resource capacity building, adoption of diagnostic imaging and pathological laboratories, early detection and treatment, surgical advancement, and adaptation of an effective cervical cancer screen. Most infection-related cancers, such as cervical, stomach, nasopharynx, and Hodgkin lymphoma, have steadily declined in Ethiopia over the last one decade.

Cancer trends and outcomes are disproportionately high in low and middle-income countries^1,3,4^. Low health care budgets^14^, overburdened health-resources with communicable diseases, child and maternal health, low universal health coverage^15^, and an increased burden of cancer all contribute to significant universal health-care disparities and inequity in low and middle-income countries. According to current evidence, primary and secondary prevention strategies could prevent more than half of all cancers^16^. A screening program based on guidelines has shown a reduction in cancer-related mortality in cervical, breast, prostate, and colorectal cancer^16^. Screening has been primarily responsible for lower rates of death and disability-adjusted life years (DALYs) for cervical cancer in Ethiopia. Currently, evidence-based modification of primary risk factors such as smoking (lung, kidney, pancreatic, and larynx cancer), H.pylori (stomach cancer), reduced alcohol consumption (liver cancer), and salted and western diets (colorectal and other GI malignancy) have aided in cancer prevention^12,17^. Global organizations advocate for and implement National Cancer Control Plans (NCCP) to address the cancer burden in low-income countries^18^. WHO leads the Global Action Plan for the Prevention and Control of NCDs 2013–2020, which aims to reduce overall mortality from cardiovascular diseases, cancer, diabetes, or chronic respiratory diseases by 25% by 2025, as well as premature mortality from noncommunicable diseases between the ages of 30 and 70^19^. Ethiopia has one cancer center that offers chemoradiotheraphy with few oncologists and radiotherapy wait times of 15–17 months and surgery wait times of 6–12 months. Ethiopia's cancer health policy in terms of prevention and control is deplorable. Ethiopia should have adopted WHO recommendations for cancer prevention, diagnosis, and management, as well as for strength national cancer control plan^3^. Low setting countries like Ethiopia should have learnt a lesson on cancer care policy development, cancer care infrastructure development, human resource capacity building, and principle of cancer prevention and control program from Rwanda^13^.

## Limitation

Although GBD studies provide qualitative and compressive evidence for policymakers, researchers, and planners, the quality and quantity of data sources available for estimation is dependent on the quality and quantity of data sources available for estimation. Cancer mortality is primarily estimated using the cancer registry, vital registration, and, to a lesser extent, other data sources. Ethiopia has only one population-based cancer registry, which only covers 3–5 percent of the total population.

## Conclusion

Overall cancer related mortality and incidence rates increased in Ethiopia. Disparities in cancer prevention, care, and control are the primary causes of these trends. Researchers, health care professionals, and policymakers must work together to develop screening guidelines and protocols, improve cancer care infrastructure, capacity building, surgical and chemoradiotheray policy, and maximize primary cancer prevention, secondary cancer prevention, early diagnosis, treatment, and rehabilitative care to reduce cancer-related mortality and disability.

## Methods

We extracted data from the GBD 2019 results too (http://ghdx.healthdata.org/gbd-results-tool). The method and data sources are described in detail in GBD 2019 publications and previous GBD publications^11,20,21^. The Guideline for Accurate and Transparent Health Estimated Reporting (GATHER) statement was used to create GBD 2019. In summary, the 2019 Global Burden of Disease, Injury, and Risk Factors Study reported national estimates of cancer incidence, mortality, and DALYs from 1990 to 2019. Estimates for GBD 2019 were analyzed and evidence for 363 non-fatal diseases, 302 deaths, and 87 risk factors were reported in 204 countries and 21 regions^11^. To calculate disease incidence, mortality, and DALYs, the GBD study collects data from vital registration, verbal autopsy, cancer registry, sample vital registry, censes, demographic and health surveys, published and unpublished health data, and other sources. GBD produced sound and up-to-date evidence of trends at the global, regional, and national levels as a result of the shift in the global agenda and increased focus on noncommunicable disease and injury. GBD studies used three main standardised modeling tools to process data, model, and generate each estimation of disease by age, location, sex, and year-Cause of Death Ensemble (CODEm), DisMod-MR, and Spatiotemporal Gaussian Process Regression (ST-GPR). Cancer registry incidence data were used to calculate the mortality rate. The first model was MIR, which is based on a cancer registry and includes both mortality and incidence. MIR is a liner-step mixed-effects model that includes a logit link function, HAQ, age, and gender as covariates. Spatiotemporal Gaussian process regression was used to smooth and adjust the final model. The final model CODEm was built using observed mortality data and MIR model estimated mortality. To estimate cancer incidence, the final cancer specific mortality estimates are divided by MRI. DisMod-MR is a Bayesian meta-regression tool that uses all available data to estimate the incidence and prevalence of each disease over time. Years lived with disability (YLDs) are calculated by dividing 10-year cancer prevalence into four categories: (1) diagnosis/treatment, (2) remission, (3) metastasis/dissemination, and (4) terminal phase. Years of life lost (YLLs) are calculated by multiplying the estimated number of deaths by age by the age's standard life expectancy. The sum of YLDs and YLLs yields disability-adjusted life-years (DALYs). For age standardised rates and all rates reported per 100,000, the GBD world population is used. All estimates have 95 percent confidence intervals (UI). The GBD2019 publications contain detailed descriptions of methodology, modeling, and data sources. The GBD2019 publications contain detailed descriptions of methodology, modeling, and data sources^11,20,21^. We focused on the national burden of cancer in Ethiopia, estimating the burden in terms of incidence, DALYs, and mortality for 29 cancer categories.

## Data Availability

Data available in GBD2019 result tool (http://ghdx.healthdata.org/gbd-results-tool).
